# Reproductive history and risk of colorectal adenocarcinoma in parous women: a Nordic population-based case–control study

**DOI:** 10.1038/bjc.2016.315

**Published:** 2016-10-04

**Authors:** Tone Bjørge, Mika Gissler, Anne Gulbech Ording, Anders Engeland, Ingrid Glimelius, Maarit Leinonen, Henrik Toft Sørensen, Steinar Tretli, Anders Ekbom, Rebecca Troisi, Tom Grotmol

**Affiliations:** 1 Department of Global Public Health and Primary Care, University of Bergen, Bergen, Norway; 2 Cancer Registry of Norway, Oslo, Norway; 3 National Institute for Health and Welfare (THL), Helsinki, Finland; 4 Department of Clinical Epidemiology, Aarhus University Hospital, Aarhus, Denmark; 5 Department of Pharmacoepidemiology, Division of Mental and Physical Health, Norwegian Institute of Public Health, Bergen/Oslo, Norway; 6 Department of Medicine, Clinical Epidemiology Unit, Karolinska Institutet and Karolinska University Hospital, Stockholm, Sweden; 7 Department of Immunology, Genetics and Pathology, Uppsala University, Uppsala, Sweden; 8 Cancer Society of Finland, Finnish Cancer Registry, Helsinki, Finland; 9 Division of Cancer Epidemiology and Genetics, National Cancer Institute, National Institutes of Health, Department of Health and Human Services, Bethesda, MD, USA

**Keywords:** colorectal cancer, reproductive history, case–control study, Nordic countries

## Abstract

**Background:**

Data are conflicting regarding the role of endogenous sex hormones in colorectal carcinogenesis. In this large population-based study, we pooled data from birth and cancer registries in four Nordic countries, to evaluate the risk of colorectal adenocarcinoma in relation to women's reproductive history.

**Methods:**

We conducted a population-based case–control study among women registered in Nordic birth registries. The study included colorectal adenocarcinoma cases diagnosed in Denmark, Finland, Norway, and Sweden during 1967–2013 and up to 10 matched controls per case, in total 22 185 cases and 220 246 controls. Odds ratios (ORs) with 95% confidence intervals (95% CIs) were derived from conditional logistic regression models. We had limited information available on possible confounders.

**Results:**

We found no evidence for associations between colorectal adenocarcinoma and parity, age at first and last birth, and time since first and last birth. The risk estimates were also close to unity for specific cancer subsites (proximal and distal colon and rectum). As well, when the analyses were stratified on menopausal status, parity, and mother's year of birth, no indication of associations was found.

**Conclusions:**

In this large, Nordic population-based study, no evidence for associations was found between women's reproductive history and colorectal adenocarcinoma in parous women.

Colorectal cancer is the second most common cancer in women worldwide (GLOBOCAN 2012), and ∼55% of the cases occur in more developed regions. In the Nordic countries during 2010–2014, the cumulative risk of disease before age 75 was 4.1% in males and 3.2% in females (NORDCAN) (Engholm *et al*, 2010). Median age at diagnosis was 69 years in males and 71 years in females. The female incidence rate was highest in Norway (36.2 per 100 000 person-years) and lowest in Finland (20.6 per 100 000 person-years).

Most cases of colorectal cancer are sporadic, and develop over many years through the adenoma–carcinoma sequence (Brenner *et al*, 2014). A family history of colorectal cancer (Taylor *et al*, 2010), inflammatory bowel disease (Ullman and Itzkowitz, 2011; Jess *et al*, 2012), smoking (Liang *et al*, 2009), high alcohol consumption (Fedirko *et al*, 2011), high consumption of red and processed meat (Bouvard *et al*, 2015), obesity (Ma *et al*, 2013), and diabetes (Larsson *et al*, 2005) are established risk factors, while physical activity (Boyle *et al*, 2012), aspirin/nonsteroidal anti-inflammatory drug (NSAID) use (Friis *et al*, 2015; Drew *et al*, 2016), and endoscopic screening, with removal of precancerous lesions (Elmunzer *et al*, 2012), have a protective effect.

As men have higher lifetime risk of colorectal cancer than women, it has been speculated that oestrogen might have a role in decreasing risk, at least partly mediated by oestrogen receptor (ER)-beta, the predominant ER subtype in the human colon (Campbell-Thompson *et al*, 2001). As opposed to the subtype ER-alpha's growth-promoting properties, ER-beta exerts an anti-proliferative effect on non-transformed colonocytes (Zhao *et al*, 2010). A protective effect of oestrogens may also be mediated through lower serum levels of insulin-like growth factor-I (Campagnoli *et al*, 2003; Renehan *et al*, 2004) and decreases in secondary bile acid production (McMichael and Potter, 1980; Bayerdorffer *et al*, 1995). Colorectal cancer subsites, which have been reported to vary by sex (Iacopetta, 2002; Lee *et al*, 2015), exhibit differences in pathogenesis, genetic and epigenetic alterations, and molecular pathways (Lee *et al*, 2015).

Strong evidence that exogenous hormones have a role in colorectal carcinogenesis derives from observational studies and clinical trials, which demonstrate an inverse relation between postmenopausal hormone therapy (HT) use and risk of colorectal cancer (Grodstein *et al*, 1999; Chlebowski *et al*, 2004; Lin *et al*, 2012). A meta-analysis, summarising results from cohort as well as case–control studies, also reported a 20% reduction in colorectal cancer risk among ever-users of oral contraceptives (OCs) compared with never-users (Bosetti *et al*, 2009).

At the same time, epidemiologic data have been conflicting regarding the role of endogenous sex hormones in colorectal carcinogenesis. A US study on the role of reproductive history and risk of colorectal cancer found positive associations between age at menopause and age at first childbirth and colorectal cancer risk in postmenopausal women, whereas age at menarche and parity were inversely associated with risk (Zervoudakis *et al*, 2011). However, a recent Swedish study found that higher parity was associated with increased risk of adenocarcinoma of the proximal colon (Lu *et al*, 2014). In contrast, a study from the European Prospective Investigation into Cancer and Nutrition found no association between reproductive history and colorectal cancer risk (Tsilidis *et al*, 2010). A 2013 meta-analysis also found no association between age at menarche and risk of colorectal cancer (Li *et al*, 2013).

In our large population-based study, pooling data from birth and cancer registries in four Nordic countries, we aimed to evaluate the overall risk of colorectal adenocarcinoma, as well as risk by cancer subsites, in relation to women's reproductive history (parity, age at first and last birth, and time since first and last birth), being a surrogate measure of her lifetime exposure to endogenous sex steroids or other biomarkers related to reproductive events.

## Materials and methods

### Data sources

This study was based on data collected from nationwide population-based registries in the four Nordic countries. Nordic birth registries contain information on all births in Denmark, Finland, Norway, and Sweden since 1973, 1987, 1967, and 1973, respectively (Gissler *et al*, 1997). Reporting of cancer cases is compulsory in the Nordic countries, and the cancer registries of Denmark, Finland, Norway, and Sweden cover the entire national populations since 1943, 1952, 1953, and 1958, respectively (Engholm *et al*, 2010). All residents of the four Nordic countries are assigned a unique country-specific personal identification number, which is used in all registries and makes accurate record linkage possible.

### Study design

The data were analysed within a case–control design, including all colorectal adenocarcinoma cases diagnosed in Denmark (1973–2011; *n*=4680), Finland (1987–2012; *n*=923), Norway (1967–2013; *n*=9797), and Sweden (1974–2013; *n*=6785) among women registered with a prior pregnancy (lasting longer than 22 weeks) in the birth registries. The rationale for including only parous women was to create a study group as homogenous as possible with respect to fertility. We included only the first cancer (any cancer) for each case. Up to 10 controls per case were sampled among women in the birth registries with a prior pregnancy lasting longer than 22 weeks, and they had to be alive and free of any cancer at the time of diagnosis of the corresponding case. Controls were matched to cases on country and birth year of the case. In total 22 185 cases and 220 246 controls were included in the study.

Colorectal adenocarcinoma cases were identified using ICD-O-3 morphology codes in Denmark, Finland and Norway, and WHO/HS/CANC/24.1 codes in Sweden. The following cancer subsites were considered: proximal colon (ICD-10/ICD-O-3 (Denmark, Finland, and Norway), C18.0–C18.5; and ICD-7 (Sweden), 153.0–153.1 and 153.4); distal colon (ICD-10/ICD-O-3, C18.6–C18.7; and ICD-7, 153.2–153.3); other colon (ICD-10/ICD-O-3, C18.8–C18.9; and ICD-7, 153.8–153.9); and rectum (ICD-10/ICD-O-3, C19.9 and C20.9; and ICD-7, 154.0).

### Statistical analysis

Odds ratios (ORs) with 95% confidence intervals (95% CIs) were derived from conditional logistic regression models. We examined the following reproductive variables: parity – number of births at the time of matching (1, 2, 3, and ⩾4); age at first birth (<20, 20–29, 30–39, and ⩾40 years); age at last birth (<20, 20–29, 30–39, and ⩾40 years); time since first birth (<10, 10–19, 20–29, and ⩾30 years); and time since last birth (<10, 10–19, 20–29, and ⩾30 years). Stratified analyses were performed for ages <50, 50–59, and ⩾60 years, which served as proxies for pre-, peri-, and postmenopausal status, respectively. We had limited information available on potential confounders.

We tested for differences between the countries by fitting logistic regression models with and without interaction terms between country of birth and the reproductive variables. The data were analysed using IBM SPSS Statistics 22 (IBM Corporation, Armonk, NY, USA) and Stata/IC 14.0 (StataCorp LP, College Station, TX, USA).

### Ethical and legal considerations

This study was approved by the ethical committees in Norway and Sweden. In Denmark, the study was approved by the Data Protection Agency. In Finland, we obtained permission to use health registry data from the National Institute for Health and Welfare after approval by the data protection authority.

## Results

Characteristics of the study population are presented in Table 1. There were only minor differences between cases and controls. Table 2 shows colorectal adenocarcinoma by subsites and country. In total, 65% of cases had colon cancer (32% in the proximal colon and 29% in the distal colon) and 35% had rectal cancer. Mean age at diagnosis was 57 years (57 years for colon cancer and 56 years for rectal cancer).

Table 3 displays ORs with 95% CIs obtained from univariate analyses for colorectal adenocarcinoma according to reproductive factors. We found no evidence for associations between colorectal adenocarcinoma and parity, age at first and last birth, and time since first and last birth. For specific subsites (proximal and distal colon and rectum) the risk estimates also were close to unity. When the analyses were stratified on menopausal status (overall and for specific subsites), parity, and mother's year of birth (<1950 and ⩾1950) (data not shown), no evidence for associations was found.

When we tested for potential differences between countries, no significantly improved fit emerged of models including interaction terms (data not shown).

## Discussion

In this large population-based Nordic study, no evidence for associations was observed between women's reproductive history and colorectal adenocarcinoma in parous women.

Studies to date regarding reproductive factors and colorectal cancer have been inconclusive. Our results showing no evidence for associations between reproductive history and colorectal adenocarcinoma, accord with some studies (Troisi *et al*, 1997; Tsilidis *et al*, 2010; Li *et al*, 2013), but not with others (Zervoudakis *et al*, 2011; Lu *et al*, 2014). A recent Swedish study, partly overlapping with the Swedish data in our study, found that higher parity was associated with increased risk of adenocarcinoma of the proximal colon, both when parous women were compared with non-parous women, and when analyses were restricted to parous women (Lu *et al*, 2014). An earlier Norwegian study, partly overlapping with the Norwegian data in our study, found associations between parity and subsites of the colorectum (Kravdal *et al*, 1993). However, in that study women with two or more children had a lower risk of cancer in the caecum or ascending colon, compared with non-parous women. A US study reported positive associations between age at first birth and colorectal cancer in postmenopausal women, whereas parity was inversely associated with risk in women with no history of HT use (Zervoudakis *et al*, 2011).

The women included in our study were relatively young as our study population was restricted to those being recorded with a prior birth in the national birth registries; 26% of cases were below age 50, and median age at diagnosis was 57 years. As median age at diagnosis of patients with genetic syndromes is relatively low, that is, in the fifth decade (Stoffel *et al*, 2009), it is possible that women with hereditary forms of colorectal cancers were overrepresented in our study population, and that these may have a weaker association with reproductive factors than sporadic forms of the disease. However, when we stratified on mother's year of birth, no difference in the risk estimates was observed.

Among the strengths of our study were its large size, inclusion of all colorectal adenocarcinoma cases (more than 22 000), with information on subsite, among women with at least one registered pregnancy in the four Nordic countries, and linkage of comprehensive databases with valid information and mandatory reporting (Teppo *et al*, 1994; Gissler *et al*, 1997; Barlow *et al*, 2009; Larsen *et al*, 2009; Gjerstorff, 2011). The study design using standardised data from registries also eliminated bias from participant self-selection and recall bias.

A study weakness was lack of information on possible confounders, such as use of exogenous hormones (OCs and HT), age at menarche and menopause, obesity, smoking, physical activity, and aspirin/NSAID use. It is possible that these factors act in opposite directions, and thus contribute to an overall null effect. However, in the Swedish study on reproductive history and colorectal adenocarcinoma, adjustment for diabetes, obesity, diagnoses associated with tobacco smoking and alcohol overconsumption, as well as bilateral oophorectomy (representing HT) did not change risk estimates materially (Lu *et al*, 2014).

A case–control design was chosen due to the strict confidentiality and data protection legislation in the Nordic countries covering access to data on the entire population and data exchange between countries. Use of 10 controls per case approximated the efficiency provided by a cohort design.

No data were missing for parity, age at last birth, and time since last birth, whereas about 48% of women lacked data on age at first birth and time since first birth, mostly due to the relatively recent establishment of the birth registries. In Finland, where the birth registry was established in 1987, more than 63% of women had missing data on age at first birth. The relatively young age of the Finnish cohort also strongly limits the number of cancer cases.

In conclusion, we found no evidence for associations between women's reproductive history (parity, age at first and last birth, and time since first and last birth) and colorectal adenocarcinoma in parous women.

## Figures and Tables

**Table 1:** Characteristics of the study population, Nordic countries, 1967–2013

	**Cases**		**Controls**	
**Variable**	***n***	**%**	***n***	**%**
**Year of birth**				
<1940	4292	19	42 860	19
1940–1949	9277	42	92 566	42
1950–1959	6059	27	60 243	27
1960–1969	2097	9	20 478	9
⩾1970	460	2	4099	2
**Age at diagnosis/matching (years)**				
<40	1549	7	14 000	6
40–49	4250	19	42 490	19
50–59	7621	34	76 125	35
60–69	6533	29	65 372	30
⩾70	2232	10	22 259	10
**Parity**				
1	2764	12	26 121	12
2	8646	39	85 954	39
3	6273	28	63 259	29
⩾4	4502	20	44 912	20
**Age at first birth (years)**				
<20	870	4	8701	4
20–29	7871	35	77 992	35
30–39	2823	13	26 395	12
⩾40	179	1	1792	1
Missing	10 442	47	105 366	48
**Age at last birth (years)**				
<20	129	1	1332	1
20–29	7992	36	78 976	36
30–39	12 481	56	124 256	56
⩾40	1583	7	15 682	7
**Time since first birth (years)**				
<10	951	4	8071	4
10–19	2315	10	22 084	10
20–29	3789	17	37 898	17
⩾30	4688	21	46 827	21
Missing	10 442	47	105 366	48
**Time since last birth (years)**				
<10	2447	11	22 158	10
10–19	4683	21	47 722	22
20–29	7275	33	73 216	33
⩾30	7780	35	77 150	35
Total	22 185	100	220 246	100

**Table 2:** Colorectal adenocarcinoma by subsite and country, Nordic countries, 1967–2013

	**Denmark (1973–2011)**		**Finland (1987–2012)**		**Norway (1967–2013)**		**Sweden (1974–2013)**		**Total (1967–2013)**	
**Subsite**	***n***	**%**	***n***	**%**	***n***	**%**	***n***	**%**	***n***	**%**
Colon										
Proximal^a^	1260	27	275	30	3492	36	2129	31	7156	32
Distal^b^	1458	31	268	29	2648	27	1949	29	6323	29
Other^c^	205	4	43	5	457	5	300	4	1005	5
Rectum^d^	1757	38	337	37	3200	33	2407	35	7701	35
Total	4680	100	923	100	9797	100	6785	100	22 185	100

a Proximal colon (ICD-10/ICD-O-3 (Denmark, Finland and Norway): C18.0–C18.5 and ICD-7 (Sweden): 153.0–153.1 and 153.4).

b Distal colon (ICD-10/ICD-O-3: C18.6–C18.7 and ICD-7: 153.2–153.3).

c Other colon (ICD-10/ICD-O-3: C18.8–C18.9 and ICD-7: 153.8–153.9).

d Rectum (ICD-10/ICD-O-3: C19.9 and C20.9 and ICD-7: 154.0).

**Table 3:** ORs^a^ with 95% CIs of colorectal adenocarcinoma according to reproductive factors and subsites, univariate analyses, Nordic countries, 1967–2013

	**Total**		**Proximal colon**		**Distal colon**		**Rectum**	
	**OR**	**95% CI**	**OR**	**95% CI**	**OR**	**95% CI**	**OR**	**95% CI**
**Age at first birth (years)**								
<20	0.98	0.91–1.06	1.01	0.88–1.16	0.90	0.78–1.04	1.04	0.92–1.18
20–29	1.00	Reference	1.00	Reference	1.00	Reference	1.00	Reference
30–39	1.08	1.02–1.14	0.99	0.89–1.09	1.15	1.04–1.27	1.07	0.98–1.17
⩾40	1.03	0.84–1.26	1.12	0.78–1.60	1.03	0.72–1.48	0.82	0.57–1.19
**Age at last birth (years)**								
<20	0.94	0.78–1.13	0.90	0.64–1.25	0.77	0.53–1.12	1.12	0.84–1.50
20–29	1.00	0.97–1.03	0.99	1.05–1.11	1.00	0.94–1.07	0.97	0.92–1.03
30–39	1.00	Reference	1.00	Reference	1.00	Reference	1.00	Reference
⩾40	1.01	0.95–1.08	0.97	0.87–1.11	1.13	1.00–1.27	0.98	0.87–1.09
**Parity**								
1	1.04	1.00–1.09	1.12	1.03–1.21	0.92	0.85–1.01	1.07	0.99–1.15
2	1.00	Reference	1.00	Reference	1.00	Reference	1.00	Reference
3	0.99	0.96–1.02	1.06	1.00–1.13	0.98	0.92–1.04	0.94	0.89–1.00
⩾4	1.00	0.96–1.04	1.01	0.94–1.09	0.99	0.92–1.07	0.98	0.92–1.05
**Time since first birth (years)**								
<10	1.00	Reference	1.00	Reference	1.00	Reference	1.00	Reference
10–19	0.94	0.84–1.04	0.84	0.69–1.02	1.00	0.83–1.21	0.94	0.79–1.12
20–29	0.85	0.77–1.07	0.78	0.62–0.98	0.86	0.69–1.06	0.97	0.79–1.18
⩾30	0.86	0.75–0.98	0.85	0.66–1.09	0.76	0.60–0.96	1.00	0.80–1.24
**Time since last birth (years)**								
<10	1.00	Reference	1.00	Reference	1.00	Reference	1.00	Reference
10–19	0.90	0.84–0.97	0.89	0.77–1.02	0.88	0.78–1.00	0.92	0.82–1.04
20–29	0.92	0.84–1.00	0.88	0.75–1.04	0.85	0.73–0.98	1.00	0.87–1.15
⩾30	0.95	0.86–1.04	0.98	0.82–1.17	0.84	0.71–1.00	1.02	0.87–1.19

Abbreviations: CI=confidence interval; OR=odds ratio.

a ORs were estimated from conditional logistic regression models, conditioned on birth year (of the case) and country.
